# Cancer of Tongue.—Removal of Entire Organ

**Published:** 1882-08

**Authors:** 


### ARTICLE X.

### *Cancer of Tongue—Removal of Entire Organ.*

N. W., aged 49, was admitted, giving the following history: Has for years indulged freely in spiritous liquors, and used the ordinary clay pipe, being an inveterate smoker. He has also been in the habit of tasting tea. No history of syphilis or suspicious ulceration of the mucous membrane of the mouth can be obtained. Some years ago he broke the first molar tooth of the right side, leaving a sharp point, which constantly irritated the side of the tongue. About the month of March, 1880, he first noticed a small pimple on the floor of the mouth, under the right border of the tongue; this was cauterized, and apparently disappeared. Some months later, a small indurated mass was felt in the situation of the pimple, and now the decayed tooth was extracted. On the 10th September, 1880, Dr. Roddick removed with scissors the nodular mass, together with a portion of the side of the tongue. From this operation the patient appeared to recover perfectly, and did not come under observation again until June 9th of this year.

On admission, the patient appeared in good health; no evidence of cachexia. On examination, a lump of about the size of a pigeon's egg is felt in the vicinity of the original induration, but involving two-thirds of the lateral half of the tongue, and interfering very much with the movements of the latter, preventing its protrusion beyond the line of the teeth. The submaxillary gland of that side is also enlarged.

*June 13th.*—The patient being etherized, and a ligature passed through the point of the tongue to control it, the operator proceeded with scissors to separate the organ from the floor of the mouth, cutting widely, especially on the right side. Nunnelly's incission was then made in the middle line, between the chin and hyoid bone; through this the chain of the ecraseur was carried and made to encircle the tongue as far back as possible, being kept in position by means of a long needle transfixing the organ. The entire tongue, with diseased portion, was then readily removed, an interval of a quarter of a minute being made to elapse between each click of the ecraseur. The hemorrhage was trifling. As a precautionary measure, a ligature was passed through the stump and held to the cheek by means of adhesive plaster. This was intended to be utilized in the event of hemorrhage or other emergency. The enlarged gland was subsequently removed by means of an incision along the margin of the jaw. A large drainage tube was passed through the opening, so as to facilitate the removal from the floor of the mouth of saliva and other discharge.

The treatment for some days consisted simply of the application of ice to the stump and the administration of small quantities of milk by means of a spoon. A mouth-wash containing carbolic acid and glycerine was used throughout.

The recovery was uninterrupted, and the patient was discharged cured on the 27th day. He was seen on the 31st of July, and could make himself readily understood, although there are many words in which th e dentals predominate, which he cannot pronounce. The disease was found, on microscopic examination, to be schirrus cancer. —*Dr. Roddick in Canada Med. and Surg. Journal.*

